# Left Ventricular Mechanics Are Associated with Short-Term Sinus Rhythm Maintenance After Electrical Cardioversion in Atrial Fibrillation

**DOI:** 10.3390/jcdd13030138

**Published:** 2026-03-13

**Authors:** Beata Uziębło-Życzkowska, Paulina Skalska, Marek Kiliszek, Małgorzata Kurpaska, Paweł Krzesiński

**Affiliations:** Department of Cardiology and Internal Diseases, Military Institute of Medicine—National Research Institute, 04-141 Warsaw, Poland; plis@wim.mil.pl (P.S.); mkiliszek@wim.mil.pl (M.K.); mkurpaska@wim.mil.pl (M.K.); pkrzesinski@wim.mil.pl (P.K.)

**Keywords:** atrial fibrillation, electrical cardioversion, left atrial function, left ventricular mechanics, myocardial work, reverse remodeling, short-term rhythm maintenance

## Abstract

(1) Background: Electrical cardioversion (ECV) is effective in restoring sinus rhythm (SR) in atrial fibrillation (AF), but the extent of atrioventricular remodeling and determinants of short-term rhythm maintenance remain unclear. This study evaluated echocardiographic changes following ECV and explored parameters associated with SR persistence. (2) Methods: We prospectively enrolled 94 patients undergoing elective ECV and performed comprehensive echocardiography before, 24 h after, and 30 days after the procedure. Rhythm status was assessed at scheduled follow-up visits. Due to the limited sample size, failure to meet the assumptions required for regression analyses, and non-normal data distributions, the analyses were primarily non-parametric and exploratory. (3) Results: Among 94 patients (mean age 65.9 +/− 9.3 years; 69% male), SR was maintained in 76 patients at 24 h and 49 patients at 30 days. Patients with sustained SR showed progressive improvement in LA reservoir strain, LA emptying fraction, and LA stiffness index, consistent with reverse atrial remodeling. Left ventricular (LV) function also improved, including LV ejection fraction, global longitudinal strain, and myocardial work indices. Between-group analyses identified several baseline LV parameters (including global wasted work, global work efficiency, LV end-systolic volume, LV end-systolic diameter, and global work index) with moderate effect sizes and possible association with short-term SR maintenance. (4) Conclusions: Successful ECV is associated with significant short-term atrioventricular functional improvement. In this exploratory single-center cohort, selected LV mechanical parameters were associated with short-term SR maintenance, while LA functional parameters mainly reflected reverse remodeling after rhythm restoration. Larger studies with longer follow-up and adjusted analyses are needed.

## 1. Introduction

Atrial fibrillation (AF) is the most prevalent sustained arrhythmia and carries significant clinical importance due to its potential complications. Treatment approaches focus on either rhythm or rate control [1]. Electrical cardioversion (ECV), alongside catheter ablation, is commonly recommended for symptomatic patients, even those on optimal pharmacological therapy, as it aims to re-establish the physiological atrial function [1]. Nevertheless, AF recurrence occurs in up to 60% of cases within the first year after cardioversion, which is thought to be closely related to the extent of atrial remodeling [2,3,4,5]. Adverse left atrial (LA) remodeling, referred to as atrial cardiomyopathy, is defined in the 2024 EHRA consensus as a set of structural, functional, and/or electrophysiological alterations affecting the LA, which may lead to clinically significant manifestations [3]. Structural remodeling of the LA primarily involves fibrotic changes within the LA walls [2,3,6]. Functional remodeling is characterized by increased stiffness and reduced compliance of the LA, resulting in impaired reservoir and pump functions [7,8]. These changes contribute to electrical remodeling, which is associated with loss of conduction homogeneity and the development of atrial arrhythmias [9].

The reversibility of LA remodeling depending on the duration of AF is a key issue in clinical electrophysiology, with important prognostic and therapeutic implications. Electrical, structural, and metabolic remodeling processes are time-dependent, meaning that the longer the arrhythmia persists, the lower the likelihood of complete or partial reversal of these changes [10]. The literature data indicate that following successful restoration of sinus rhythm (SR) through ECV, partial reversal of adverse structural and functional changes in the LA may occur, a phenomenon referred to as reverse (beneficial) remodeling. Preliminary studies suggest that effective termination of AF and maintenance of SR are associated with a reduction in LA size, improvement in its mechanical function (contractility and filling), limitation of fibrosis progression, and stabilization of electrical parameters [11,12,13].

Nevertheless, such improvement is not observed in all patients. In some individuals, the LA remains enlarged, or its function may deteriorate due to advanced and potentially irreversible structural changes [10]. The likelihood and magnitude of reverse remodeling appear to depend more on AF chronicity (overall duration), baseline atrial substrate, and comorbidity burden than on AF typology alone.

Despite growing interest in this topic, it remains unclear whether, and in which patients, reverse remodeling of the LA occurs after successful ECV, and whether baseline echocardiographic parameters can discriminate patients who will maintain SR in the short term. Therefore, this study aimed to explore the structural and functional response of the left atrium and left ventricle (LV) to restoration of SR in patients with AF and to identify echocardiographic parameters associated with short-term maintenance of SR.

## 2. Methods

Patients admitted for elective ECV between January 2022 and June 2023 were prospectively enrolled in the study as part of the HOPE-AF project (Hemodynamic Profile of Patients with PErsistent Atrial Fibrillation—Assessment of Its Relation to Physical Efficiency and Effectiveness of Electrical Cardioversion through Hemodynamic Changes Related to Restoring Sinus Rhythm). This was an exploratory prospective single-center cohort study. A formal a priori sample size calculation was not performed because the study was designed as a pilot project. Therefore, all consecutive eligible patients during the recruitment period were included.

Inclusion criteria required a duration of AF of ≤12 months and successful ECV with maintenance of SR for at least 24 h.

Exclusion criteria were: poorly controlled arterial hypertension (blood pressure at admission >180/100 mmHg); coronary artery disease; LV ejection fraction (LVEF) <40%; severe valvular heart disease or prior surgical intervention for valvular dysfunction; severe pulmonary diseases (COPD stage C or D, uncontrolled asthma, pulmonary hypertension, pulmonary embolism); chronic kidney disease stage G4 or G5 according to KDIGO; severe systemic inflammatory disease; obesity with BMI > 40 kg/m^2^; severe psychiatric disorders; history of pacemaker or other implantable device; estimated life expectancy <12 months; musculoskeletal disorders significantly limiting mobility; and lack of patient consent. These criteria were selected to reduce major non-AF determinants of hemodynamics and myocardial mechanics, improve the feasibility of repeated high-quality echocardiographic assessment, and increase internal consistency of this exploratory cohort. However, they limit generalizability and may introduce selection bias.

Outcome definition and rhythm assessment: The immediate outcome was maintenance of SR at 24 h after ECV. Short-term maintenance was defined as SR at the scheduled 30-day follow-up visit. AF recurrence was defined as documented AF on follow-up ECG and/or clinically documented recurrence during routine care. The short follow-up duration should be interpreted as an early remodeling endpoint, not long-term rhythm-control success.

A comprehensive transthoracic echocardiography (TTE) was performed on the day of cardioversion and repeated 24 h and 30 days after successful ECV. The follow-up echocardiographic examinations were performed only in patients who maintained sinus rhythm. The 30-day follow-up was chosen to assess early reverse remodeling after SR restoration in a prospective pilot design and was not intended to represent long-term rhythm-control efficacy.

All standard left- and right-chamber measurements were performed in accordance with current chamber quantification and deformation imaging recommendations. For detailed evaluation of LA and LV function, left atrial strain (LAS) was assessed, including reservoir strain (LASr) in all patients, and additionally conduit strain (LAScd) and contraction strain (LASct) in patients with successful cardioversion only. Left atrial emptying fraction (LAEF) was calculated using dedicated automated software for LA assessment. The left atrial stiffness index (LASI) was defined as E/e’ divided by LASr. The contraction strain index (CSI) was calculated as (LASct/LASr) × 100.

All 2D and speckle-tracking echocardiographic parameters were acquired based on current guidelines and consensus documents for chamber quantification, deformation imaging, and echocardiographic reporting [14,15,16].

Ethnicity and medication: Patients were recruited from a single-center Polish referral population, and the cohort was relatively homogeneous ethnically.

### 2.1. Ethical Statement

The study was conducted in accordance with Good Clinical Practice guidelines and the Declaration of Helsinki. The study protocol was approved by the local ethics committee (Resolution No. 30/WIM/2019), and written informed consent was obtained from all study participants.

### 2.2. Statistical Analysis

Statistical analyses were performed as exploratory analyses. Due to the modest sample size, limited number of outcome events (especially at 30 days), and non-normal distribution of multiple variables, the primary analyses were non-parametric and focused on associations rather than formal predictive modeling.

To assess changes in echocardiographic parameters over time, separate analyses were conducted for the two primary time points (24 h and 30 days). Repeated-measures ANOVA and mixed models were not applied because of missing data, group imbalances after rhythm recurrence, and violations of model assumptions. For variables with three repeated measures, the Friedman test was used. If significant (*p* < 0.05), post hoc pairwise comparisons were performed using the Wilcoxon signed-rank test with Bonferroni correction (*p* < 0.017). For variables measured twice in the same patients, the Wilcoxon signed-rank test was applied; for comparisons between independent groups, the Mann–Whitney U test was used.

Effect sizes were calculated for each test (eta-squared for Mann–Whitney, Kendall’s W for Friedman, and r for Wilcoxon) and interpreted as measures of association magnitude and practical significance rather than evidence of discrimination. Spearman’s rank correlation was used to assess associations between variables. Descriptive statistics included median and interquartile range (Q1–Q3). A *p* value < 0.05 was considered statistically significant.

Follow-up echocardiographic analyses were performed only in patients who maintained sinus rhythm (SR). Therefore, unavailable follow-up measurements were not considered typical missing data in the strict sense but were mainly related to rhythm recurrence, which precluded assessment of SR-dependent parameters (e.g., LASct). Additional unavailable measurements were due to occasional feasibility or image-quality limitations. Consequently, the follow-up sample represented a selected subgroup rather than a fully representative continuation of the baseline cohort, which may introduce selection (survivorship) bias, particularly in reverse-remodeling analyses restricted to patients maintaining SR.

## 3. Results

**Figure 1:**
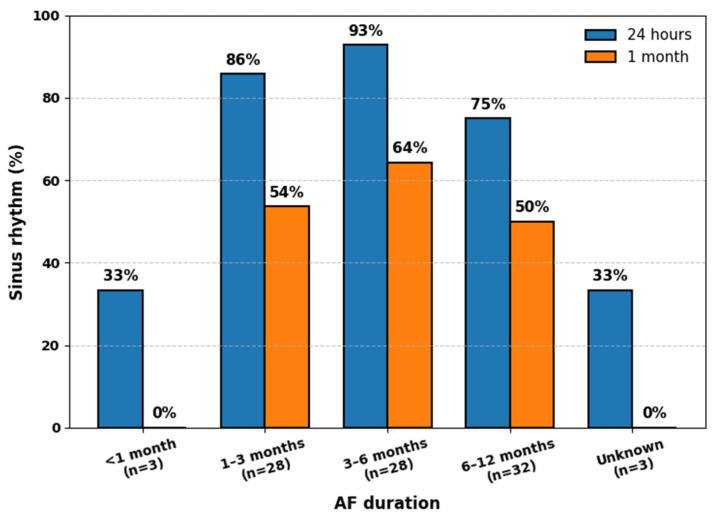
Percentage of patients maintaining sinus rhythm 24 h and 1 month after ECV, depending on atrial fibrillation duration. SR, sinus rhythm; AF, atrial fibrillation; ECV, Electrical cardioversion.

Among the initially enrolled cohort of 94 patients (mean age 65.9 ± 9.3 years), 69% were male, and 42.6% were classified as obese (BMI ≥ 30 kg/m^2^). Regarding the duration of AF, approximately one-third of patients (34%, 32 individuals) had AF lasting between 6 and 12 months, and 34% (32 patients) had a short AF duration of less than 3 months. Following ECV, SR was successfully maintained in 76 patients (80.9%) at 24 h. At the 30-day follow-up, SR persisted in 49 patients, corresponding to 52% of the study population. Figure 1 illustrates the percentage of successful ECV by AF duration. Two patients were lost to follow-up at the 30-day mark.

Baseline pharmacotherapy, including antiarrhythmic and rate-control drugs, is reported in Supplementary Table S1. We compared medication distribution between groups with and without recurrence at 24 h and at 30 days. In our cohort, medication use was generally balanced between groups. The only significant between-group difference was beta-blocker use in the 24 h analysis (70% vs. 91.9%, *p* = 0.02). No significant differences were observed for antiarrhythmic/rate-control medications in the 30-day recurrence analysis (including beta-blockers, amiodarone, and digoxin). Therefore, although we acknowledge that pharmacotherapy may act as a confounder, the observed medication profile does not suggest a major systematic imbalance across most treatment classes, particularly for the 30-day endpoint.

The most prevalent comorbidities observed in this patient group included hypertension, diagnosed in 79.8% of cases, and heart failure with preserved and mildly reduced LVEF, which was present in 28.7% of patients. Additionally, diabetes mellitus was identified in 28.7% of the cohort.

Baseline echocardiographic parameters indicated a mean LA area of 29.8 ± 5.5 cm^2^ and a mean indexed left atrial volume (LAVI) of 48.9 ± 12.8 mL/m^2^, reflecting marked LA enlargement in the study population, which may influence the likelihood of maintaining SR after ECV.

### 3.1. Echocardiographic Changes Following Successful Cardioversion

Among patients who maintained SR following successful ECV, significant and progressive improvements were observed in a range of echocardiographic parameters reflecting both LA and LV function. These changes were evident as early as 24 h post-procedure and became more pronounced by the 30-day follow-up. Improvements were observed in global atrioventricular strain (GAVS) and LASr, both of which are sensitive markers of LA compliance and reservoir function. Correspondingly, the E/LASr ratio, a surrogate of LV filling pressure, showed a significant decline, indicating reduced LA afterload. Additionally, LAEF increased, and the LASI decreased, further supporting the observation of improved LA mechanical function and reduced diastolic burden.

Left ventricular function also benefited from the restoration and maintenance of SR. Notably, there was a measurable increase in LVEF and enhancement of myocardial contractility as reflected by improved global longitudinal strain (GLS LV) and global work index (GWI). All changes in echocardiographic parameters reflecting structural and functional remodeling of the LA and LV during the follow-up period are summarized in Table 1.

Importantly, parameters that can only be assessed during SR—such as LASct, contraction strain index (CSI), transmitral A wave velocity, E/A ratio, and tissue Doppler-derived lateral A’ wave velocity—also showed substantial improvement between 24 h and 30 days post-ECV. Each of these parameters increased significantly over time, reflecting progressive recovery of LA contractile function and improved atrioventricular interaction during SR maintenance. Detailed data for these variables are presented in Table 2.

### 3.2. Comparative Analysis Between Patients with and Without Maintained Sinus Rhythm (Association Analyses)

**Figure 2:**
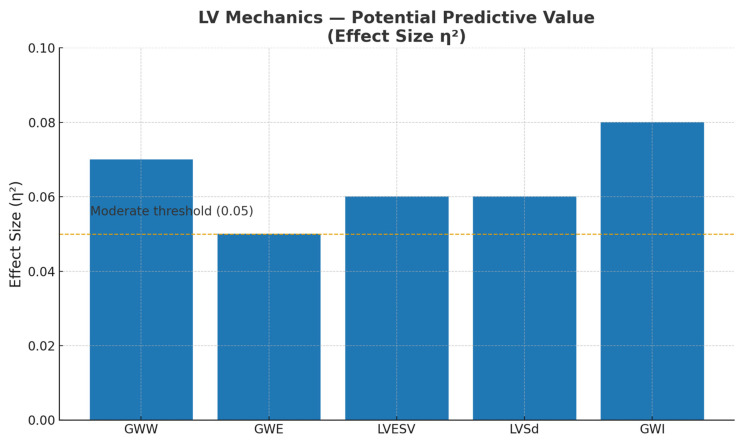
Effect size (η^2^) values for LV mechanics parameters associated with the potential predictive value for sinus rhythm maintenance after ECV. A moderate effect size threshold was considered at η^2^ ≥ 0.05. LVESV, left ventricle end-systolic volume; LVSd, left ventricle end-systolic diameter; GWE, global work efficiency; GWI, global work index; GWW, global wasted work; ECV, Electrical cardioversion.

In between-group analyses (patients with vs. without maintained SR), only a few baseline LV parameters showed moderate effect sizes (eta-squared = 0.05–0.08), suggesting limited but potentially meaningful associations with short-term cardioversion success. For distinguishing between groups in the immediate post-cardioversion period (24 h), the differentiating parameters were global wasted work (GWW) and global work efficiency (GWE), both with moderate effect sizes (eta-squared = 0.05–0.06). For short-term SR maintenance at 30 days, the key associated parameters were LV end-systolic volume (LVESV), LV end-systolic diameter (LVSd), and GWI, with effect sizes ranging from eta-squared = 0.06 to 0.08 (Figure 2). Detailed results of this comparative analysis are summarized in Supplementary Tables S2 and S3. These findings should be interpreted as association signals requiring validation in larger cohorts with adjusted models.

Several parameters—including LASr, E/LASr, GAVS, LASct, CSI, A wave, lateral A’ wave, and E/A ratio—demonstrated the most substantial changes following ECV, with effect sizes (W or r) ≥0.7. These findings may indicate reverse atrioventricular remodeling. Although these variables did not show predictive value at baseline, their significant post-ECV dynamics may carry clinical importance and merit further investigation (see Table 1 and Table 2).

## 4. Discussion

We have shown that successful electrical cardioversion is associated with measurable and sustained short-term changes in both LA and LV structure and function. Among the assessed parameters, selected LV mechanical variables were associated with short-term SR maintenance in unadjusted analyses, whereas LA function parameters were not associated with short-term outcome at baseline.

Electrical cardioversion of AF is a rather simple procedure that leads to the fast restoration of sinus rhythm. Although it has been known for years, only a few studies have investigated electrical cardioversion and its mechanical consequences [17]. ECV remains a clinically relevant rhythm-control option for selected symptomatic patients with AF, especially when rapid restoration of sinus rhythm is needed [1].

With the increasing ability of echocardiography to assess parameters describing the left ventricle and left atrium (myocardial work, atrial and ventricular strains), we decided to perform a study evaluating numerous echocardiographic parameters before and after electrical cardioversion. However, because AF recurrence after ECV is common and ECV is often only one step in a broader rhythm-control strategy, our study was designed to assess the early hemodynamic and mechanical echocardiographic response after successful ECV, rather than to compare ECV with ablation or surgery.

We observed that left atrial functional parameters (LASr, LASct, LASI, and E/A ratio) often remain abnormal shortly after ECV but show marked improvement within one month. This pattern is consistent with previous studies, which found that atrial strain rate measured immediately after ECV was significantly lower than in control patients but improved over the first month [12]. Other reports have shown that successful ECV restores LASr, reduces LA volume, and improves LVEF [18,19]. In line with these findings, we also demonstrate improvement in all left ventricular parameters after ECV, including LV strains, GWI, and LVEF.

Interestingly, among the many parameters evaluated for association with electrical cardioversion outcome, only those related to LV function were significant in unadjusted between-group comparisons. Although we assessed numerous LA parameters, we did not observe baseline associations with short-term SR maintenance, in contrast to several previous studies [20,21,22]. Possible explanations include the short follow-up period, the modest sample size and event count, heterogeneity of AF chronicity, and the lack of adjusted analyses.

When evaluating the 24 h success of electrical cardioversion, the differentiating parameters (with moderate effect sizes) were global wasted work (GWW) and global work efficiency (GWE). Specifically, lower GWW and higher GWE were associated with a greater likelihood of maintaining sinus rhythm after 24 h. This finding aligns with the definitions of these parameters, as GWE is inversely related to GWW [23]. With global constructive work (GCW) remaining unchanged, it is the changes in GWW that primarily influence our results. Abnormalities in GWW and GWE have been observed in patients with LV remodeling [24], and these abnormalities tend to increase with higher cardiovascular risk [23]. These parameters have rarely been studied in patients with AF; however, a small study demonstrated that patients with AF have lower global work index (GWI), GCW, and GWE, and higher GWW, with more pronounced effects in persistent compared to paroxysmal AF [25].

When assessing factors associated with successful cardioversion at one month, left ventricular parameters again proved important: higher LV end-systolic volume (LVESV), larger LV end-systolic diameter (LVSd), and lower global work index (GWI) were associated with an increased risk of AF recurrence. Notably, most patients in our cohort did not have significant LV dysfunction, making this finding particularly interesting. This observation highlights the potential role of heart failure with preserved ejection fraction in predicting rhythm maintenance after AF electrical cardioversion. Previous studies have shown that patients with larger LVSd are at increased risk of AF progression from paroxysmal to persistent, even after AF ablation [26].

### Strengths and Limitations

We present data from a prospective single-center study of consecutive patients with AF undergoing ECV, with comprehensive echocardiographic assessment including advanced strain and myocardial work parameters. The study documents early reverse atrioventricular remodeling after restoration of SR. However, the limitations are important: single-center design, modest cohort size, lack of formal a priori power analysis, short follow-up (30 days), and absence of standardized continuous rhythm surveillance (e.g., systematic Holter monitoring). A key limitation of this study is the lack of multivariable adjustment. We explored multivariable regression models; however, diagnostic assessment showed important assumption violations, including heteroscedasticity and non-random residual patterns, which indicated poor model fit and limited reliability of the estimated coefficients. Therefore, we used more robust non-parametric methods, supplemented by effect size estimates (η^2^). As a result, residual confounding cannot be excluded, including the possible influence of factors such as antiarrhythmic therapy. Thus, the findings should be interpreted as associative rather than evidence of independent effects. The statistical strategy and its limitations were additionally reviewed by an independent biostatistician.

Accordingly, the findings should be considered hypothesis-generating.

## 5. Conclusions

In conclusion, effective electrical cardioversion results in quantifiable short-term alterations in left atrial and left ventricular structure and function. In this exploratory cohort, selected LV mechanics parameters were associated with short-term SR maintenance, whereas LA function parameters mainly reflected reverse remodeling after SR restoration and did not show significant baseline associations with short-term outcome. Larger prospective studies with longer follow-up, standardized rhythm monitoring, medication-adjusted analyses, and multivariable models are needed before clinical predictive use can be inferred.

## Figures and Tables

**Table 1:** Significant changes in echocardiographic parameters assessed prospectively before, 24 h after, and 30 days after successful cardioversion.

Variable	Baseline (Median [IQR])	24 h After ECV (Median [IQR])	30 Days After ECV (Median [IQR])	*p* Value	Kendall’s W Coefficient *
Left atrial parameters					
LASr (%)	10 (8–12)	15 (10–18)	20 (15–25)	<0.001	0.83
LASI	0.96 (0.66–1.25)	0.69 (0.46–1.16)	0.47 (0.28–0.63)	<0.001	0.59
LAEF (%)	28 (22–32)	31 (24–43)	47 (37–50)	<0.001	0.60
LAVI (ml/m^2^)	46.2 (40.1-56.8)	49.2 (42.0-56.1)	41.7 (34.8-50.7)	0.002	0.19
E/LASr	7.44 (6.3–10.3)	5.33 (3.9–8.4)	2.73 (2–4.5)	<0.001	0.75
Left ventricular parameters					
GLS LV(%)	14.2 (11.8–16)	16.7 (15–18.9)	18.8 (16.7–19.9)	<0.001	0.59
GWI (mmHg%)	1334 (1059–1532)	1528 (1276–1842)	1853 (1508–2124)	<0.001	0.52
LVEF (%)	50 (46–56.5)	57 (49.5–62)	60 (57–65)	<0.001	0.62
GAVS (%)	24.6 (19.9–27.3)	30.4 (26.7–35.1)	38.7 (32.6–43.6)	<0.001	0.87

Abbreviations: E/LASr, E-to-left atrial reservoir strain ratio; GAVS, global atrio-ventricular longitudinal strain (GLS LV+LASr); GLS LV, left ventricular global longitudinal strain; GWI, global work index; LAEF, left atrial emptying fraction; LASI, LA stiffness index (E/e’/LASr); LASr, left atrial reservoir strain; LV EF, left ventricular ejection fraction; LAVI, left atrial volume index; ECV, Electrical cardioversion. * (≥0.5 for strong correlation and ≥0.7 for very strong correlation).

**Table 2:** Significant changes in echocardiographic parameters assessed prospectively during sinus rhythm—24 h and 30 days after successful cardioversion.

Variable	24 h After ECV (Median [IQR])	30 Days After ECV (Median [IQR])	*p* Value	Effect Size *r* *
LASct (%)	4 (2–7)	11 (7–13)	<0.001	0.88
CSI	28 (20–36.8)	53.3 (41.7–60)	<0.001	0.86
A wave (cm/s)	32 (26–45)	63 (47–72)	<0.001	0.87
E/A ratio	2.4 (1.4–3.1)	0.9 (0.7–1.3)	<0.001	0.87
a’ lateral (cm/s)	4 (3–6)	8 (7–10)	<0.001	0.88

Abbreviations: a’ lateral, the lateral mitral annular late-diastolic velocity; A wave, the late-diastolic mitral inflow velocity; E/A ratio, ratio of early- to late-diastolic transmitral flow velocities; CSI, contraction strain index (LASct/LASr); LASct, left atrial contraction strain; ECV, Electrical cardioversion. * (>0.5 for large effect size).

## Data Availability

The data presented in this study are available on request from the corresponding author.

## Supplementary Materials

The following supporting information can be downloaded at https://www.mdpi.com/article/10.3390/jcdd13030138/s1. Table S1. Baseline pharmacotherapy in the study group, including antiarrhythmic and rate-control drugs; Table S2. Results of the statistical analysis of between-group differences (with vs. without maintained SR) in the context of immediate cardioversion success (assessed at 24 h); Table S3. Results of the statistical analysis of between-group differences (with vs. without maintained SR) in the context of long-term cardioversion success (assessed at 1 month).
