# Supplementary material for: Left Ventricular Mechanics Are Associated with Short-Term Sinus Rhythm Maintenance After Electrical Cardioversion in Atrial Fibrillation
Source: J Cardiovasc Dev Dis. 2026 Mar 13;13(3):138. doi: 10.3390/jcdd13030138 (PMC13026168; doi:10.3390/jcdd13030138)
Supplement: Supplementary file 1 [file jcdd-13-00138-s001.zip › jcdd-4073962-supplementary.pdf]

Table S1. Baseline pharmacotherapy in the study group, including antiarrhythmic and rate-control drugs.

Variable	Patients with AF	Patients with SR	p value
Assessment at 24hours after ECV			
ACEI	9 (45)	39 (52.7)	0.62
ARB	3 (15)	15 (20.3)	0.75
beta-blockers	14 (70)	68 (91.9)	0.02
MRA	3 (15)	18 (24.3)	0.55
diuretuk	11 (55)	42 (56.8)	0.99
amiodarone	4 (20)	9 (12.2)	0.46
digoxin	0 (0)	3 (4.1)	0.26
SGLT2 inhibitors	1 (5)	9 (12.2)	0.68
Ca blockers	4 (20)	24 (32.4)	0.41
statins	12 (60)	44 (59.5)	0.96
Assessment at 30 days after ECV			
ACEI	22 (48.9)	26 (55.3)	0.68
ARB	7 (15.6)	10 (21.3)	0.59
beta-blockers	36 (80)	44 (93.6)	0.07
MRA	9 (20)	12 (25.5)	0.62
diuretuk	27 (60)	25 (53.2)	0.54
amiodarone	8 (17.8)	5 (10.6)	0.38
digoxin	0 (0)	3 (6.4)	0.24
SGLT2 inhibitors	4 (8.9)	6 (12.8)	0.74
Ca blockers	11 (24.4)	16 (34)	0.36
statins	27 (60)	27 (57.5)	0.83

Abbreviations: ACEI, Angiotensin-Converting Enzyme Inhibitor;
AF, atrial fibrillation; ARB, Angiotensin Receptor Blocker; ECV, electrical cardioversion; MRA, Mineralocorticoid Receptor Antagonist; SR, sinus rhythm.
Data presented as n (%)

Table S2. Results of the statistical analysis of between-group differences (with vs. without maintained SR) in the context of immediate cardioversion success (assessed at 24 hours).

Variables	p value	η² **
Age (years)	U* =516; p = 0.11	NS
BMI (kg/m²)	U =563.5; p = 0.25	NS
	Left and right atrial parameters	
LAd (cm)	U =578; p = 0.34	NS
LASr (%)	U =510; p = 0.83	NS
LASI	U =393.5; p = 0.36	NS
LA area (cm²)	U =590.5; p = 0.41	NS
RA area (cm²)	U =664.5; p = 0.99	NS
LAV (ml)	U =505; p = 0.72	NS
LAVI (ml/m²)	U =565; p = 0.75	NS
LAEF (%)	U =501; p = 0.75	NS
E/LASr	U =506; p = 0.8	NS
	Left and right ventricle parameters	
LVEDV (ml)	U =328; p = 0.23	NS
LVESV (ml)	U =316; p = 0.17	NS
LV SV (ml)	U =379; p = 0.63	NS
GLS LV (%)	U =396; p = 0.97	NS
GWI (mmHg%)	U =380; p = 0.78	NS
GCW (mmHg%)	U =333; p = 0.34	NS
GWW (mmHg%)	U =255; p = 0.04	0.06
GWE (%)	U =263.5; p = 0.049	0.05
GAVS (GLS+LASr) (%)	U =371; p = 0.69	NS
e'avg (cm/s)	U =486; p = 0.9	NS
E/e' avg	U =367.5; p = 0.21	NS
E (cm/s)	U =468.5; p = 0.96	NS
LV EF (%)	U =575.5; p = 0.37	NS
RVd (cm)	U =582.5; p = 0.41	NS
IVSd (cm)	U =565; p = 0.28	NS
LVDd (cm)	U =662.,5; p = 0.9	NS
LVSd (cm)	U =593; p = 0.64	NS
LVMI (g/m²)	U =383; p = 0.09	NS

Abbreviations: BMI, body mass index; E, mitral early-diastolic inflow peak velocity; E/LASr, E-to-left atrial reservoir strain ratio; GAVS, global atrio-ventricular longitudinal strain (GLS LV+LASr); GCW, global constructive work; GLS LV, left ventricular global longitudinal strain; GWE, global work efficiency; GWI, global work index; IVSd, interventricular septum diameter in diastole; LA area, left atrial area; LAd, left atrial diameter; LAEF, left atrial emptying fraction; LASI, LA stiffness index (E/e'/LASr); LASr, left atrial reservoir strain; LAV, left atrial volume; LAVI, left atrial volume index; LVDd, left ventricular end-diastolic diameter; LVSd, left ventricular end-systolic diameter; LVEDV, left ventricular end-diastolic volume; LV EF, left ventricular ejection fraction; LVESV, left ventricular end-systolic volume; LVMI left ventricular mass index; LV SV, left ventricular stroke volume; RA area, right atrial area; RVd, right ventricular diameter.

*U — Mann–Whitney U test statistic (non-parametric between-group comparison)
** η² — effect size measure (eta-squared)

Table S3. Results of the statistical analysis of between-group differences (with vs. without maintained SR) in the context of long-term cardioversion success (assessed at 1 month).

Variables	p	η^2 **
Age (years)	U* = 950; p = 0.53	NS
BMI (kg/m^2)	U = 909; p = 0.34	NS
Left and right atrial parameters		
LAd 1 (cm)	U = 849.5; p = 0.21	NS
LAd 2 (cm)	U = 403; p = 0.34	NS
LASr 1 (%)	U = 622; p = 0.13	NS
LASr 2 (%)	U = 343; p = 03	NS
LASI 1	U = 595; p = 0.4	NS
LASI 2	U = 360.5; p = 0.75	NS
LA area 1 (cm^2)	U = 961; p = 0.73	NS
LA area 2 (cm^2)	U = 439.5; p = 0.99	NS
RA area 1 (cm^2)	U = 885; p = 0.43	NS
RA area 2 (cm^2)	U = 341.5; p = 0.23	NS
LAV 1 (ml)	U = 740.5; p = 0.62	NS
LAV 2 (ml)	U = 406; p = 0.96	NS
LAVI 1 (ml/m^2)	U = 849; p = 0.82	NS
LAVI 2 (ml/m^2)	U = 363; p = 0.78	NS
LAEF 1 (%)	U = 624.5; p = 0.14	NS
LAEF 2 (%)	U = 363.5; p = 0.48	NS
E/LASr 1	U = 733; p = 0.69	NS
E/LASr 2	U = 328.5; p = 0.21	NS
LASct 2 (%)	U = 293.5; p = 0.12	NS
LAScd 2 (%)	U = 385; p = 0.94	NS
CSI 2	U = 302.5; p = 0.16	NS
Left and right ventricle parameters		
LVEDV 1 (ml)	U = 520.5; p = 0.29	NS
LVEDV 2 (ml)	U = 345; p = 0.59	NS
LVESV 1 (ml)	U = 438; p = 0.04	0.06
LVESV 2 (ml)	U = 340; p = 0.53	NS
LVSV 1 (ml)	U = 605; p = 0.95	NS
LVSV 2 (ml)	U = 369; p = 0.88	NS
GLS LV 1 (%)	U = 526.5; p = 0.54	NS
GLS LV 2 (%)	U = 314.5; p = 0.37	NS
GWI 1 (mmHg%)	U = 484; p = 0.26	NS
GWI 2 (mmHg%)	U = 236; p = 0.04	0.08
GCW 1 (mmHg%)	U = 503; p = 0.37	NS
GCW 2 (mmHg%)	U = 253.5; p = 0.07	NS
GWW 1 (mmHg%)	U = 485; p = 0.26	NS
GWW 2 (mmHg%)	U = 345; p = 0.84	NS
GWE 1 (%)	U = 526; p = 0.54	NS
GWE 2 (%)	U = 312.5; p = 0.44	NS
GAVS 1 (%)	U = 543; p = 0.69	NS
GAVS 2 (%)	U = 334; p = 0.69	NS
e'avg 1 (cm/s)	U = 668; p = 0.79	NS
e'avg 2 (cm/s)	U = 319; p = 0.32	NS
E/e' avg 1	U = 541; p = 0.15	NS
E/e' avg 2	U = 358.5; p = 0.73	NS
E 1 (cm/s)	U = 666; p = 0.77	NS
E 2 (cm/s)	U = 338.5; p = 0.34	NS
EF 1 (%)	U = 773.5; p = 0.09	NS
EF 2 (%)	U = 386; p = 0.51	NS
A 2 (cm/s)	U = 313; p = 0.34	NS
E/A 2	U = 297.5; p = 0.23	NS

a'm 2 (cm/s)	U = 279; p = 0.12	NS
a'l 2 (cm/s)	U = 293.5; p = 0.19	NS
RVd 1 (cm)	U = 955; p = 0.84	NS
RVd 2 (cm)	U = 464,5; p = 0.92	NS
IVSd 1 (cm)	U = 862; p = 0.24	NS
IVSd 2 (cm)	U = 395.5; p = 0.28	NS
LVDd 1 (cm)	U = 908; p = 0.43	NS
LVDd 2 (cm)	U = 379; p = 0.19	NS
LVSd 1 (cm)	U = 862.5; p = 0.64	NS
LVSd 2 (cm)	U = 295; p = 0.02	0.08
LVMI 1 (g/m^2)	U = 747; p = 0.79	NS
LVMI 2 (g/m^2)	U = 327.5; p = 0.32	NS

Abbreviations: 1- measurements in baseline (before cardioversion); 2- measurements 24 hours post cardioversion; BMI, body mass index; E, mitral early-diastolic inflow peak velocity; E/LASr, E-to-left atrial reservoir strain ratio; GAVS, global atrio-ventricular longitudinal strain (GLS LV+LASr); GCW, global constructive work; GLS LV, left ventricular global longitudinal strain; GWE, global work efficiency; GWI, global work index; IVSd, interventricular septum diameter in diastole; LA area, left atrial area; LAd, left atrial diameter; LAEF, left atrial emptying fraction; LASI, LA stiffness index (E/e'/LASr); LASr, left atrial reservoir strain; LAV, left atrial volume; LAVI, left atrial volume index; LVDd, left ventricular end-diastolic diameter; LVSd, left ventricular end-systolic diameter; LVEDV, left ventricular end-diastolic volume; LV EF, left ventricular ejection fraction; LVESV, left ventricular end-systolic volume; LVMI left ventricular mass index; LV SV, left ventricular stroke volume; RA area, right atrial area; RVd, right ventricular diameter.

*U — Mann–Whitney U test statistic (non-parametric between-group comparison)
** η^2 — effect size measure (eta-squared)
